# Combined treatment with allogeneic Epstein–Barr- and human polyomavirus 1 specific T-cells in progressive multifocal leukoencephalopathy and EBV infection: a case report

**DOI:** 10.1177/17562864241253917

**Published:** 2024-05-28

**Authors:** Sandra Nay, Nora Möhn, Lea Grote-Levi, Agnes Bonifacius, Mieke L. Saßmann, Kevin Karacondi, Sabine Tischer-Zimmermann, Henning Pöter, Nima Mahmoudi, Mike P. Wattjes, Britta Maecker-Kolhoff, Günter Höglinger, Britta Eiz-Vesper, Thomas Skripuletz

**Affiliations:** Department of Neurology, Hannover Medical School, Hannover, Germany; Department of Neurology, Hannover Medical School, Hannover, Germany; Department of Neurology, Hannover Medical School, Hannover, Germany; Institute of Transfusion Medicine and Transplant Engineering, Hannover Medical School, Hannover, Germany; Department of Neurology, Hannover Medical School, Hannover, Germany; Department of Neurology, Hannover Medical School, Hannover, Germany; Department of Pediatric Hematology and Oncology, Hannover Medical School, Hannover, Germany; Department of Neurology, Hannover Medical School, Hannover, Germany; Department of Diagnostic and Interventional Neuroradiology, Hannover Medical School, Hannover, Germany; Department of Neuroradiology, Charité Berlin, Corporate Member of Freie Universität zu Berlin, Humboldt-Universität zu Berlin, Berlin, Germany; Department of Diagnostic and Interventional Neuroradiology, Hannover Medical School, Hannover, Germany; Department of Neuroradiology, Charité Berlin, Corporate Member of Freie Universität zu Berlin, Humboldt-Universität zu Berlin, Berlin, Germany; Department of Pediatric Hematology and Oncology, Hannover Medical School, Hannover, Germany; German Center for Infection Research, Hannover, Germany; Department of Neurology, LMU University Hospital, Ludwig-Maximilians-Universität München, Munich, Germany; German Center for Neurodegenerative Diseases, Munich, Germany; Munich Cluster for Systems Neurology, Munich, Germany; Department of Neurology, Hannover Medical School, Hannover, Germany; Institute of Transfusion Medicine and Transplant Engineering, Hannover Medical School, Hannover, Germany; German Center for Infection Research, Hannover, Germany; Department of Neurology, Hannover Medical School, Carl-Neuberg-Straße 1, Hannover 30625, Germany; Centre for Individualised Infection Medicine, Hannover, Germany

**Keywords:** allogenic T-cells, BKV, Epstein–Barr virus, human immunodeficiency virus, JCV, progressive multifocal leukoencephalopathy

## Abstract

Opportunistic viral infections in individuals with severe immunodeficiency can lead to fatal conditions such as progressive multifocal leukoencephalopathy (PML), for which treatment options are limited. These infections pose significant risks, especially when co-infections with other viruses occur. We describe a combined therapy approach using directly isolated allogeneic Human Polyomavirus 1 (also known as BKV) and Epstein–Barr virus (EBV) specific cytotoxic T-cells for the treatment of PML in conjunction with identified EBV in the cerebrospinal fluid (CSF) of a male patient infected with human immunodeficiency virus (HIV). A 53-year-old HIV-positive male, recently diagnosed with PML, presented with rapidly worsening symptoms, including ataxia, tetraparesis, dysarthria, and dysphagia, leading to respiratory failure. The patient developed PML even after commencing highly active antiretroviral therapy (HAART) 3 months prior. Brain magnetic resonance imaging (MRI) revealed multifocal demyelination lesions involving the posterior fossa and right thalamus suggestive of PML. In addition to the detection of human polyomavirus 2 (also known as JCV), analysis of CSF showed positive results for EBV deoxyribonucleic acid (DNA). His neurological condition markedly deteriorated over the following 2 months. Based on MRI, there was no evidence of Immune Reconstitution Inflammatory Syndrome contributing to this decline. The patient did not have endogenous virus-specific T-cells. We initiated an allogeneic, partially human leukocyte antigen-matched transfer of EBV and utilizing the cross-reactivity between BKV and JCV–BKV specific T-cells. This intervention led to notable neurological improvement and partial resolution of the MRI lesions within 6 weeks. Our case of a patient with acquired immune deficiency syndrome demonstrates that PML and concurrent EBV co-infection can still occur despite undergoing HAART treatment. This innovative experimental therapy, involving a combination of virus-specific T-cells, was demonstrated to be an effective treatment option in this patient.

## Introduction

Progressive multifocal leukoencephalopathy (PML) is an opportunistic infection of the brain caused by the human polyomavirus 2, commonly referred to as JC virus (JCV) or JC polyomavirus. This serious infection almost exclusively affects patients with severe cellular immunodeficiency, either from related underlying diseases or previous use of immunosuppressive therapies.^1^ According to current Center of Disease Control guidelines for patients infected with human immunodeficiency virus (HIV), PML is categorized as one of the diseases that define acquired immunodeficiency syndrome (AIDS).^2^

Before the widespread use of highly active antiretroviral therapy (HAART), PML was more common among HIV patients, and outcomes were poorer than today.^3^ Although HIV-related PML is considered to have a somewhat less severe outcome compared to other conditions, it still carries a substantial death rate, with about half of the patients not surviving past a year.^4^ Until recently, there were no effective treatment options for this often-fatal disease. Within the last years, the use of anti-PD-1 (programmed cell death protein 1) antibodies (immune checkpoint inhibitor, ICI),^5–20^ and the application of allogeneic virus-specific T-cells^21–29^ have shown encouraging treatment results for some PML patients. Notably, the absence of virus-specific T-lymphocytes in the blood has been identified as a negative prognostic factor in HIV-positive PML-patients.^4^

Patients with AIDS are not only susceptible to polyomavirus infections but also to other opportunistic pathogens, often experiencing multiple infections simultaneously.^30^ Infection or reactivation with the Epstein–Barr virus (EBV) are particularly prominent in this context. A recent meta-analysis revealed that EBV is detectable in the cerebrospinal fluid (CSF) of approximately 20% of HIV-positive patients without lymphoma.^31^ Similarly to JCV, in situations of cellular immunodeficiency, EBV can shift into an intensified replication state.^32^ In addition to possibly causing symptomatic infections such as encephalitis, meningitis, and myelitis, EBV is also suspected to have a key role in the development of lymphoproliferative diseases.^32^ Currently, there is no standardized treatment for symptomatic EBV infections.^33^ A promising experimental strategy for transplant recipients who have developed EBV-related post-transplant lymphoproliferative disorders (PTLDs) involves transfer of EBV specific T-cells.^34,35^

In a few immunosuppressed patients, the use of virus-specific T-cells has been proven effective against various viruses.^36–39^ Due to the structural homology of the two viruses JCV and BKV, which belong to the polyomavirus family, not only JCV-specific but also BKV-specific T-cells have been used in PML, taking advantage of this cross-reactivity. However, the combined use of these T-cells targeting both JCV or BKV and EBV has not been published. We present a case involving a patient with AIDS who had severe PML and a simultaneous EBV infection, and who responded positively to treatment with virus-specific T-cells targeting both viruses.

## Case presentation

**Figure 1.**
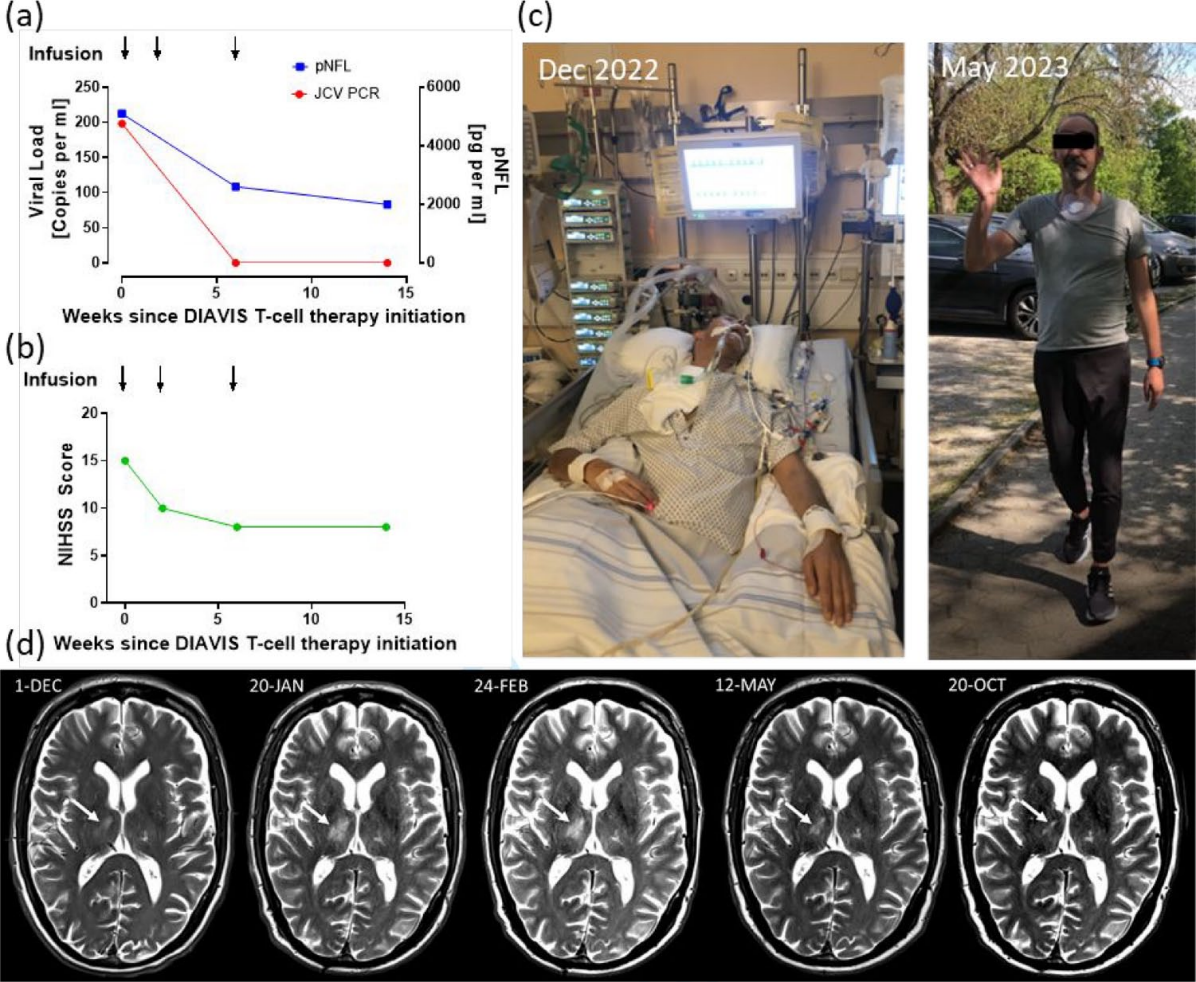
Clinical and laboratory follow-up parameters. (a) JCV viral load (red) and pNFL concentration (blue) on multiple time points since therapy. Black arrows: Times of DIAVIS T-cell infusion. (b) NIHSS score on multiple time points since therapy initiation. Black arrows: Times of DIAVIS T-cell infusion. The score was determined retrospectively on the basis of available neurological examination results. (c) These photographs show the severe clinical condition of the patient, with the left image taken before the initiation of DIAVIS BKV and EBV T-cell treatment, and the right image captured 19 weeks following the commencement of therapy. The photographs were taken by the patient’s relatives. We have obtained the patient’s consent to share these photographs. (d) T2*-weighted MRI image before, during, and after therapy initiation. Despite initiation of HAART, the MRI revealed PML progression with enlarging lesions prior to therapy initiation, consistent with the clinical deterioration. After initiation of therapy with DIAVIS BKV and EBV T-cells in mid-January 2023, the lesions showed a steady regression. DIAVIS T-cells, directly isolated allogeneic virus-specific T-cells; HAART, highly active antiretroviral therapy; JCV, human polyomavirus 2; MRI, magnetic resonance imaging; NIHSS, National Institutes of Health Stroke Scale; PML, progressive multifocal leukoencephalopathy; pNFL, plasma neurofilament light chain.

A 53-year-old man was diagnosed with HIV in July 2022 and subsequently began HAART treatment. By November 2022, he started experiencing progressive neurological symptoms. His initial complaints included diplopia and nuclear facial palsy, leading to hospitalization. The brain magnetic resonance imaging (MRI) revealed multifocal T2-hyperintense and partial T1-hypointense lesions involving the bilateral mesencephalon, pons, right thalamus, and left cerebellum suggestive of PML. Contrast enhancement suggestive of active immune response, such as in the context of Immune Reconstitution Inflammatory Syndrome (IRIS), was not observed.^40^ CSF analysis indicated a positive PCR (Polymerase Chain Reaction) result for JCV (3818 copies/ml) and detected EBV deoxyribonucleic acid (DNA) within the CSF below the limit of quantification (LOQ). When the patient was admitted to our hospital, approximately 2 months after the onset of neurological symptoms, he had already undergone a percutaneous endoscopic gastrostomy tube insertion due to significant dysphagia, and a tracheostomy following respiratory failure worsened by aspiration pneumonia, during which atypical mycobacteriosis was detected. In addition to the primary symptoms, neurological examination showed tetraparesis with a more pronounced effect on the arms, especially the left, and a deviation of the uvula. The patient was unable to sit or move on his own. With the support of therapists on either side, he was able to stand and take a few steps, displaying marked ataxia in both limbs and trunk. At the same time, MRI also showed progression of PML with enlargement of PML-typical parenchymal changes, particularly in the thalami and midbrain [Figure 1(d)]. MRI features suggestive of IRIS were still absent. Despite starting HAART 5 months prior to admission, no BKV or JCV specific T lymphocytes were detectable in his peripheral blood. Virus-specific T-cell monitoring was performed using INFγ ELISpot assay (interferon gamma enzyme-linked immunosorbent spot assay). The CD4+ T-lymphocyte count had marginally increased from 96 to 103 cells/µl, with a concurrent reduction in HIV RNA to below LOQ.

In a compassionate use setting of an experimental therapy, we treated the patient with directly isolated allogeneic T-cells (DIAVIS T-cells) targeting BKV and EBV from a partially human leukocyte antigen (HLA)-matched unrelated donor (6/10, Table 1). Over a period of 6 weeks, the patient received three infusions: the first was a fresh dose containing 2.0 × 10e4 CD3+ T-cells per kilogram of body weight (week 0), followed by two cryopreserved doses each with 2.5 × 10e4 CD3+ T-cells per kilogram of body weight (weeks 2 and 6). The production process is detailed in prior publications.^23,25^ Prior to manufacturing, the frequencies of BKV and EBV specific T cells were determined to identify a suitable T-cell donor. This was done using the IFN-γ Cytokine Secretion Assay, which is largely analogous to clinical-grade manufacturing.^41^ Here, the determined frequencies of BKV and EBV specific T-cells were 0.02% and 0.07% CD3+/IFN-γ+, respectively, and purities after magnetic enrichment were 23.2% and 47.8%, respectively. In brief, DIAVIS T-cells were obtained from leukapheresis product in a multi-step manufacturing process. After stimulation of the cells with MACS GMP PepTivators BKV_VP1, BKV_LT, EBV_EBNA1, and EBV_Select in combination (1 µg/ml per peptide, Miltenyi Biotec, Bergisch Gladbach, Germany), IFNγ capture was used to enrich IFNγ+ T cells. The purity of the obtained T-cell product was 42.8% CD3+/IFNγ+.

**Table 1.** HLA typing of patient and T-cell donor.

	HLA-A		HLA-B		HLA-C		HLA-DR		HLA-DQ	
Patient	24:02	32:01	44:02	51:01	07:04	01:02	11:01	13:01	03:01	06:03
T-cell donor	24:02	32:01	44:02	27:05	05:01	01:02	04:01	13:01	03:02	06:03

HLA, human leukocyte antigen.

Six weeks following the commencement of treatment, significant neurological improvements were observed in the patient [Figure 1(c)]. His tetra-ataxia lessened, enabling him to operate a smartphone and write clearly. The tracheostomy was successfully removed, and he began transitioning to oral feeding. Notable improvements were also seen in his dysarthria and facial palsy, and the deviation of his uvula resolved. However, oculomotor deficits remained, such as limited abduction in the left eye and double vision when looking to the left. The infusions of DIAVIS BKV and EBV T-cells were well-tolerated and did not result in any adverse effects. Figure 1 and Table 2 display the clinical and laboratory results throughout the course of treatment.

**Table 2.** Routine CSF follow-up results.

Time since DIAVIS T-cell therapy initiation (weeks)	0	4	14
CSF cells/µl	1	1	3
CSF lactate (mmol/l)	1.76	1.94	1.98
CSF protein (mg/l)	340	310	440
Oligoclonal bands	Type 3	Type 3	Type 3
Albumin quotient	3.81	5.17	6.37
Intrathecal IgM synthesis (%)	47	27.4	0
Intrathecal IgG synthesis (%)	61.1	47.9	38.6
Intrathecal IgA synthesis (%)	0	26.8	0

Follow-up routine CSF parameters from the time of therapy initiation are shown here. No new inflammatory process, such as pleocytosis indicative of PML-IRIS, was observed.

CSF, cerebrospinal fluid; DIAVIS T-cells, directly isolated allogeneic virus-specific T-cells; Ig, immunoglobulin; IRIS, Immune Reconstitution Inflammatory Syndrome; PML, progressive multifocal leukoencephalopathy.

Eighteen weeks post-treatment, the patient’s oculomotor function had normalized, with improvements in left-sided facial palsy and ataxia, though mild issues remained. Proximal weakness persisted in his left leg, but he could move independently and walk over 100 m [Figure 1(b)]. Brain MRI indicated partial resolution of T2-hyperintensities particularly in the right thalamic area without new lesions [Figure 1(d)]. EBV was undetectable in the CSF, and no JCV DNA was found after therapy initiation [Figure 1(a)]. A small pool of BKV and EBV specific T-cells was present as early as 2 weeks after therapy initiation in the patient’s blood, as evaluated by qualitative IFNγ ELISpot assay. No BKV specific T-cells were detected after 6 and 14 weeks, but endogenous JCV-specific T-cells could now be detected at a higher frequency.

## Discussion

In this report, we highlight the innovative use of combined directly isolated allogenic BKV and EBV specific T-cells for treating PML patients who also have a concurrent EBV infection. Following transfer of BKV and EBV DIAVIS T-cells, the patient showed considerable clinical improvement and a decrease in leukoencephalopathy, as verified by MRI. Notably, no side effects were observed, and there was no indication of IRIS. To our knowledge, this represents the second case of an HIV-associated PML being treated with allogenic T-cells, and more significantly, it is the first use of a multivirus-targeted DIAVIS T-cell strategy in a patient with HIV.

While AIDS is the primary cause of PML, accounting for about 50% of all diagnoses, it is paradoxically underrepresented in terms of promising treatments for this condition.^39^ Although there are no approved medications, experimental treatments involving ICIs and the application of virus specific allogenic T-cells have been utilized in treatment.^7–14,20–26,28,29,39^ However, out of 91 PML patients treated with ICI, only 12 were related to AIDS.^20^ Within this subgroup, 6 out of 12 patients died within a year, with causes including PML progression, PML-induced IRIS, and a combination of PML with other contributing diseases. This mortality rate mirrors the historical mortality for HIV-associated PML without ICI treatment.^4^ However, this data may not be comprehensive enough due to potential biases in patient selection. It’s likely that patients showing PML progression despite established HAART are more frequently chosen for ICI treatment. Furthermore, as indicated by previous studies, ICI may not be optimal in cases lacking endogenous virus-specific T-cells.^5,6,25^ Considering this, we believe that our choice of DIAVIS T-cell infusion over ICI therapy in this case, guided by an extensive pre-treatment evaluation of the patient’s immune status, played a critical role in the successful outcome.

Out of 34 documented cases of PML treated with various cellular therapies, only one involved an HIV-positive patient who had been on HAART for 4 months prior to the initial transfer of BKV specific T-cells.^24^ While this patient finally showed clinical improvement, he initially experienced symptoms of PML–IRIS, including a temporary clinical worsening in the first 2 weeks of therapy. In contrast, our case showed no adverse effects, and there were no signs of IRIS. Besides concerns about IRIS, there have been recent concerns regarding virus specific T-cell therapy in HIV patients, particularly about the potential migration of HIV into the central nervous system along with the allogenic T-cells.^42^ However, this did not occur in our patient’s case. While the therapy was well tolerated in our case, it is important to carefully consider potential risks. Experience from larger cohorts will be necessary to accurately assess the side effect profile.

In our patient, DIAVIS T-cells targeting EBV were also employed. EBV specific T-cells have been applied in treating refractory PTLD in transplant recipients and selected immunodeficiency patients.^34,41^ Despite the elevated risk of EBV-associated lymphoma in HIV-positive individuals, this treatment approach has not yet been extended to HIV-positive patients. In scenarios involving severe immunosuppression among HIV-negative individuals, a small number of patients have received treatment with multivirus-specific T-cells, targeting a range of viruses including EBV, cytomegalovirus, human herpesvirus 6, BKV, and adenovirus.^36,38,43^ This strategy, however, has not yet been expended to PML patients with co-infections or HIV-positive individuals. Furthermore, there’s ongoing discussion about whether application of virus specific T-cells, especially in HIV-positive patients, could be used as a preventative measure to reduce the risk of malignancies associated with opportunistic oncogenic viruses.^34^ This preventative concept has also been suggested for managing PTLD in transplant recipients, underlining its potential applicability in various contexts.

The specific contribution of each therapy to our patient’s recovery is difficult to ascertain due to the continued use of HAART. Considering that the patient developed PML despite starting HAART 3 months earlier and continued to deteriorate in the following 2 months, it seems unlikely that HAART alone would have led to recovery. Following the DIAVIS T-cell therapy, there was significant improvement within 6 weeks, transitioning the patient from a nearly palliative condition to an independent walking capability. It’s crucial to emphasize that these observations are derived from the experience of a single patient, and therefore, any resulting recommendations should be made with caution. Nevertheless, for AIDS patients who develop PML despite antiviral HIV therapy, we suggest the consideration of additional DIAVIS T-cell therapy as a treatment option.

## Declarations

**Ethics approval and consent to participate:** The therapy was conducted in a compassionate use setting as an experimental therapy after patient’s informed consent to the therapy approach. Because the therapy was applied as consented compassionate use, it did not require an ethical board approval.

**Consent for publication:** We received the patient’s written informed consent for publication of this case report.

**Competing interests:** The authors declare no conflict of interest. Outside the submitted work, the authors received honoraria for lectures, travel grants, or research grants. Günter Höglinger has ongoing research collaborations with Roche, UCB, Abbvie; serves as a consultant for Abbvie, Alzprotect, Amylyx, Aprineua, Asceneuron, Bayer, Bial, Biogen, Biohaven, Epidarex, Ferrer, Kyowa Kirin, Lundbeck, Novartis, Retrotope, Roche, Sanofi, Servier, Takeda, Teva, UCB; received honoraria for scientific presentations from Abbvie, Bayer, Bial, Biogen, Bristol Myers Squibb, Kyowa Kirin, Pfizer, Roche, Teva, UCB, Zambon; holds a patent on Treatment of Synucleinopathies (US 10,918,628 B2, EP 17 787 904.6-1109/3 525 788); received publication royalties from Academic Press, Kohlhammer, and Thieme. Thomas Skripuletz reports research support from Alnylam Pharmaceuticals, CSL Behring, Novartis; honoraria for lectures and travel expenses for attending meetings from Alexion, Alnylam Pharmaceuticals, argenx, Bayer Vital, Biogen, Bristol Myers Squibb, Celgene, Centogene, CSL Behring, Euroimmun, Grifols, Hexal AG, Janssen-Cilag, Merck Serono, Novartis, Pfizer, Roche, Sanofi, Siemens, Swedish Orphan Biovitrum, Teva, Viatris; consultant fees from Alexion, Alnylam Pharmaceuticals, Biogen, Centogene, CSL Behring, Grifols, Hexal AG, Janssen-Cilag, Merck Serono, Novartis, Roche, Sanofi, Swedish Orphan Biovitrum, Viatris.

**Availability of data and materials:** Not applicable.
